# COVID‐19 y disparidades por género y renta: evidencia de Filipinas

**DOI:** 10.1111/ilrs.12227

**Published:** 2022-02-22

**Authors:** Rouselle F. LAVADO, Keiko NOWACKA, David A. RAITZER, Yana van der Meulen RODGERS, Joseph E. ZVEGLICH

**Affiliations:** ^1^ Organización Mundial de la Salud (en el momento de redactar el artículo, Banco Asiático de Desarrollo); ^2^ Banco Asiático de Desarrollo; ^3^ Universidad de Rutgers

**Keywords:** COVID‐19, brecha de género, empleo, trabajo no remunerado, pobreza, confinamiento, mujeres, Filipinas, personal docente

## Abstract

La pandemia de COVID‐19 y las políticas de contención asociadas han afectado más a Filipinas que a la mayoría de los países en desarrollo por aplicar confinamientos de los más estrictos del mundo, y cierres generales de escuelas de los más prolongados. Con un modelo de simulación novedoso, se estiman los efectos de estos factores por sector, región administrativa y género en los niveles de empleo, ingresos y pobreza, con particular atención al empleo de los docentes y a los ingresos de las personas con hijos pequeños en relación con el cierre de escuelas. Se constata que la pandemia tiene repercusiones sin precedentes en la actividad económica y afecta desproporcionadamente a las mujeres.

### 1. Introducción: COVID-19 y género

La pandemia de COVID-19 ha provocado inmensas pérdidas en términos de salud y bienestar de las personas, los hogares y las sociedades de todo el mundo. La creciente evidencia de una disparidad de género en lo que respecta a la tasa de letalidad de la COVID-19 en todos los países indica una desventaja para los hombres, pero el diferencial de género se invierte en relación con otras dimensiones no fisiológicas de la enfermedad (Bahn, Cohen y Rodgers 2020; Wenham, Smith y Morgan 2020). Resulta especialmente preocupante la sobre-

* Organización Mundial de la Salud; lavador@who.int (en el momento de redactar el artículo, Banco Asiático de Desarrollo). ** Banco Asiático de Desarrollo; knowacka@adb.org, draitzer@adb.org y jezveglich@adb.org. *** Universidad de Rutgers; yana.rodgers@rutgers.edu (autora para la correspondencia). Los autores desean agradecer a Glenita Amoranto, Liming Chen y Xylee Javier sus útiles aportaciones para el ejercicio de simulación. También desean agradecer a Esther Barrett y a Yasuyuki Sawada y a los participantes en el Foro de Economistas del Banco Asiático de Desarrollo de 2021 sus comentarios sobre un borrador anterior. Las opiniones expresadas en el presente artículo corresponden a sus autores y no reflejan necesariamente los puntos de vista o las políticas del Banco Asiático de Desarrollo, ni de su junta directiva, ni de los gobiernos en ellos representados.

La responsabilidad de las opiniones expresadas en los artículos solo incumbe a sus autores, y su publicación en la *Revista Internacional del Trabajo* no significa que la OIT las suscriba.

Artículo original: «COVID-19 disparities by gender and income: Evidence from the Philippines», *International Labour Review* 161 (1). Traducción del equipo editorial de la Revista. Traducido también al francés en *Revue internationale du Travail* 161 (1).

[Correction added on 26 March 2022, after first online publication: The copyright line was changed.]

rrepresentación de las mujeres entre los trabajadores con salarios bajos que se encuentran en primera línea con respecto al riesgo de exposición al virus. Las mujeres también han experimentado pérdidas de empleo relativamente mayores en muchos países, dado que el comercio minorista, la alimentación y la hotelería se encuentran entre los sectores más afectados y las mujeres están desproporcionadamente representadas en ellos (Alon et al. 2020; OIT 2021). Un creciente volumen de evidencia mundial indica que el aumento del trabajo de cuidados durante la pandemia ha recaído de forma desproporcionada sobre las mujeres (Craig y Churchill 2021; İlkkaracan y Memiş 2021; Zamarro y Prados 2021). También se ha obtenido evidencia de que los trabajadores de bajos ingresos y con empleos precarios y sus familias están soportando relativamente más los costes de los confinamientos en el hogar en todos los países (Kabeer, Razavi y Rodgers 2021). Los trabajadores y trabajadoras migrantes se han convertido en un grupo especialmente vulnerable en diversos contextos, pues su situación se ve a menudo agravada por las prohibiciones de viajar, su exclusión de los regímenes de asistencia pública de emergencia, la precariedad de las viviendas que ocupan, las violaciones de sus derechos laborales y las restricciones a la inmigración (Guadagno 2020; Koh 2020; Stevano et al. 2021).

Entre los países en desarrollo, Filipinas es uno de los más afectados. Los datos mundiales sobre las infecciones por COVID-19 indican que Filipinas tiene más casos confirmados en laboratorio y más muertes per cápita que muchos otros países de Asia, siendo el país del sudeste asiático con más casos diagnosticados per cápita. En su punto álgido, en abril de 2021, Filipinas tenía 1351 casos confirmados quincenalmente por millón de habitantes, lo que superaba tanto la media mundial de 1215 como el número de casos de cualquier país de Asia, excepto la India.[1] Al igual que en la mayoría de los países, la morbilidad y la mortalidad por COVID-19 en Filipinas han afectado más a los hombres que a las mujeres: en abril de 2021, el 53 por ciento de los casos confirmados de COVID-19 y el 60 por ciento de los fallecidos eran hombres, en comparación con los promedios mundiales del 54 por ciento de casos y el 59 por ciento de muertes.[2] Sin embargo, según la evidencia que se está obteniendo, la diferencia de género se invierte en cuanto a la carga impuesta por las políticas de contención, especialmente en lo que respecta a la pérdida de empleo de las mujeres, la carga de trabajo de cuidados no remunerado relativamente mayor en el hogar, la salud mental y la violencia doméstica (ONU Mujeres y Women Count 2020; Cho et al. 2021; De los Santos y Labrague 2021).

Al igual que muchos otros gobiernos asiáticos, el Gobierno de Filipinas impuso el confinamiento a mediados de marzo de 2020. Fue uno de los más estrictos y largos del mundo (Hapal 2021). Incluyó el cierre de todas las escuelas hasta que se lograra la vacunación generalizada de la población (lo que podría

[1] Hannah Ritchie, Edouard Mathieu, Lucas Rodés-Guirao, Cameron Appel, Charlie Giattino, Esteban Ortiz-Ospina, Joe Hasell et al., base de datos en línea «Coronavirus Pandemic (COVID-19)». https://ourworldindata.org/coronavirus (consultada en abril de 2021).

[2] Global Health 50/50, «The COVID-19 Sex-Disaggregated Data Tracker», base de datos del Proyecto Sexo, Género y COVID-19. https://globalhealth5050.org/the-sex-gender-and-covid-19-project/ (consultada en abril de 2021).

no ser posible hasta dentro de varios años), mientras que los niños de la mayoría de los demás países volvieron a la escuela a finales de 2020 (Raitzer et al. 2020). Filipinas representa un importante estudio de caso, no solo porque es uno de los países en desarrollo más afectados y con un confinamiento más prolongado, sino también porque se considera un líder en materia de igualdad de género en la región, con sólidas leyes al respecto y en un lugar destacado de las clasificaciones relativas a varios indicadores clave de igualdad de género, como los salarios, la educación y la participación política.

El presente artículo explora las repercusiones de la COVID-19 en Filipinas analizando el impacto diferenciado del confinamiento según el género, la región administrativa y el grupo de ingresos. Para ello utilizamos un novedoso modelo de simulación basado en la Encuesta de Población Activa (Labour Force Survey, LFS) y en la Encuesta de Ingresos y Gastos Familiares (Family Income and Expenditure Survey, FIES) de Filipinas que nos permite estimar el efecto de la pandemia y de las políticas de confinamiento en los niveles de empleo, ingresos y pobreza, y cómo dicho efecto difiere según el género y la región administrativa. El análisis incluye un estudio de caso centrado en las consecuencias adicionales del cierre de las escuelas en el mercado laboral, en términos de pérdidas tanto de empleo en el caso de los docentes de escuelas privadas como de ingresos en el caso de los progenitores que han tenido que ocuparse del cuidado de los niños y de la educación en casa. Comprobamos que la crisis de la COVID-19 y las políticas de confinamiento asociadas han tenido consecuencias sin precedentes para la actividad económica en Filipinas y han afectado de forma desproporcionada a las mujeres. Nuestro estudio podría proporcionar datos para elaborar políticas con perspectiva de género que pongan el debido énfasis en la igualdad de género y en la reducción de la pobreza.

El resto del artículo está organizado como sigue. En la segunda sección se presenta el contexto filipino. En la tercera se expone el modelo de simulación que aplicamos para estimar los impactos diferenciales de la pandemia y de las políticas de contención, y en la cuarta presentamos nuestros resultados. La quinta sección se centra específicamente en los efectos del cierre de escuelas sobre el empleo de los docentes y los ingresos de los hogares, y la sexta concluye resumiendo nuestros resultados y algunas recomendaciones políticas de ellos derivadas.

### 2. Contexto de Filipinas

Filipinas suele ser uno de los países asiáticos mejor clasificados en relación con varios indicadores clave de igualdad de género, como los relativos a los salarios, el nivel educativo y la participación política. En 2019, por ejemplo, el Foro Económico Mundial (FEM) situó a Filipinas en 14.ª posición entre 153 países en cuanto a participación y oportunidades económicas, y en la 29.ª en cuanto a participación política, superando a países de la región con mayores niveles de ingresos, como el Japón, la República de Corea y Singapur (FEM 2019). Una prueba del nivel relativamente alto de participación política femenina es que el 28 por ciento de los miembros del parlamento son mujeres, frente a una media mundial del 25 por ciento y una media regional del 20 por ciento (PNUD 2020).

Posiblemente en respuesta al relativamente severo confinamiento impuesto, el país descendió en ambas clasificaciones del FEM hasta los puestos 18.º y 33.º, respectivamente, según el informe de 2021 (FEM 2021). Filipinas también ha logrado la paridad de género en cuanto a nivel educativo, siendo uno de los pocos países de ingresos bajos y medianos en los que las mujeres superan a los hombres en la mayoría de las cohortes en cuanto al nivel de educación formal obtenido. Además, una proporción relativamente alta de empleadas remuneradas filipinas tiene estudios universitarios en comparación con otros países con niveles de desarrollo comparables, y el país destaca también por haber alcanzado casi la paridad de ingresos entre los géneros, una rareza a nivel mundial, independientemente del nivel de renta nacional (BAsD 2015; Zveglich, Rodgers y Laviña 2019).

La Magna Carta of Women (Carta Magna de las Mujeres) de 2008 está ampliamente reconocida como un instrumento jurídico histórico para la lucha contra la discriminación, la protección de los derechos humanos de las mujeres y la promoción de la igualdad de género.[3] Los notables avances mencionados son el resultado de medidas políticas proactivas y del activismo de la sociedad civil en un contexto de fuerte crecimiento económico. Sin embargo, a pesar de estos esfuerzos y logros en materia salarial, política y educativa, Filipinas se queda atrás en otros indicadores clave, lo que explica su relativamente mediocre clasificación, en el puesto 104.º, del Índice de Desigualdad de Género del Programa de las Naciones Unidas para el Desarrollo (PNUD 2020). Según este índice, Filipinas sale peor parada en cuanto a la situación de la salud reproductiva de las mujeres, reflejo de una tasa de mortalidad materna y de una tasa de natalidad de adolescentes relativamente altas, así como una tasa de participación laboral femenina bastante baja en comparación con los países vecinos e incluso con la media mundial (ibíd.).

La pandemia de COVID-19 constituye, por tanto, una importante prueba de la resistencia de los progresos realizados para cerrar la brecha de género en relación con varios indicadores clave de empoderamiento, así como de su solidez frente a una perturbación socioeconómica tan abrumadora. También pone a prueba hasta qué punto el país se ha quedado atrás en otras áreas, especialmente en lo que respecta a la participación laboral de las mujeres y a la salud reproductiva. A escala mundial ya se tiene conciencia de que la pandemia representa un riesgo considerable de perder los avances en materia de igualdad de género, pues según las predicciones en el momento de redactar el presente artículo, se perderán décadas de progreso en este ámbito. Aunque la morbilidad femenina debida a la COVID-19 sigue siendo inferior a la de los hombres, las mujeres y las niñas se han visto desproporcionadamente afectadas por las políticas de contención. La OIT calculó en 2020 que la pérdida de puestos de trabajo para las mujeres sería mayor que para los hombres; en efecto, 527 millones de mujeres en todo el mundo (es decir, el 41 por ciento del empleo femenino total) trabajan en sectores vulnerables muy afectados, en comparación con solo el 35 por ciento del empleo masculino total (OIT 2020).[4] También se previó que, debido al cierre

[3] Republic Act No. 9710, *Official Gazette*, 14 de agosto de 2009.

[4] Se trata de sectores como la hotelería y la alimentación, los servicios inmobiliarios, empresariales y administrativos, la industria manufacturera y el comercio mayorista y minorista.

de escuelas, 11 millones de niñas en todo el mundo quedarían desescolarizadas (UNESCO 2020). El cierre de guarderías y escuelas ha incrementado la cantidad de tiempo que las mujeres dedican a las actividades de cuidados, exacerbando la distribución desigual de este tipo de trabajo no remunerado dentro del hogar (en Asia y el Pacífico, las mujeres dedicaban en promedio cuatro veces más tiempo al trabajo de cuidados no remunerado que los hombres (OIT 2019)). También se esperaba que las tasas de mortalidad materna e infantil aumentaran en todo el mundo debido a las interrupciones en los servicios de salud, con estimaciones de 56 700 muertes maternas adicionales en 118 países de ingresos bajos y medianos (Roberton et al. 2020). Los índices de violencia en la pareja también han aumentado en todo el mundo, en relación con las medidas de confinamiento y el estrés social y económico. En toda Asia y el Pacífico, los medios de comunicación locales y las organizaciones de la sociedad civil señalaron aumentos del volumen de llamadas a las líneas telefónicas de atención frente a la violencia doméstica de hasta más del doble, así como en el número de mujeres que intentaban acceder a refugios destinados a víctimas de violencia doméstica en comparación con cifras anteriores a la COVID-19 (CESPAP 2020).

Según las estadísticas disponibles, Filipinas también está sufriendo estas tendencias mundiales. El Grupo de Trabajo Interinstitucional para la Gestión de las Enfermedades Infecciosas Emergentes de Filipinas[5] anuncia sus medidas de contención en términos de niveles de «cuarentena comunitaria», que incluyen restricciones específicas por sector sobre el número de empleados permitidos en los lugares de trabajo. Los niveles de cuarentena también varían geográficamente, de forma que las restricciones más estrictas se aplican en las principales áreas metropolitanas, donde se han producido más casos de COVID-19. El nivel más estricto se denomina «cuarentena comunitaria reforzada» (*enhanced community quarantine*) y consiste en general en la obligación de permanencia en el hogar. Bajo este nivel de cuarentena, se espera que la población permanezca en sus casas, excepto para realizar tareas esenciales (como la compra de alimentos o medicinas), y solo se permite acudir a los lugares de trabajo a los empleados de los servicios esenciales. El nivel de «cuarentena comunitaria general» *(general community quarantine)*, iniciado en mayo de 2020, es menos estricto y permite el transporte público con aforo reducido, así como las operaciones comerciales con un número limitado de trabajadores y clientes. Por último, la «cuarentena comunitaria general modificada» (*modified general community quarantine*) es el régimen menos estricto y permite que la mayoría de las empresas funcionen en zonas con pocos casos de COVID-19.

Los datos agregados publicados, basados en la LFS filipina, indican que el desempleo aumentó del 5,2 por ciento en abril de 2019 al 17,6 por ciento en abril de 2020, cuando se decretaron los cierres de empresas, y que los hombres representan una mayor proporción de los desempleados. La participación laboral se redujo del 61,3 al 55,7 por ciento durante el mismo periodo, y las mujeres son mayoría entre las personas económicamente inactivas (PSA 2020). Además, según las encuestas, las mujeres filipinas sufrieron más efectos psicológicos ad-

[5] Covid-19 Inter-Agency Task Force for The Management of Emerging Infectious Diseases Resolutions (https://doh.gov.ph/COVID-19/IATF-Resolutions).

versos por la pandemia, por ejemplo, niveles más altos de estrés, ansiedad y depresión (Tee et al. 2020). La pandemia también contribuyó a los problemas y disparidades en el hogar. La violencia doméstica, que ya era un problema de larga data en Filipinas, también aumentó bajo los estrictos protocolos de confinamiento (Sediri et al. 2020). Además, la carga de trabajo en el hogar se incrementó y recayó en mayor medida en las mujeres. Gran parte de ese aumento de la carga de trabajo consistió en el cuidado de los niños y en su escolarización en el hogar, especialmente en las zonas remotas con escasa conectividad a internet donde la interacción con los profesores no era posible (Cho et al. 2021). Según datos de una encuesta realizada en los hogares filipinos, el 40 por ciento de las mujeres experimentaron un incremento del tiempo dedicado en el hogar a tres tareas como mínimo relacionadas con el trabajo doméstico no remunerado debido a la pandemia, en comparación con el 32 por ciento de los hombres (ONU Mujeres y Women Count 2020). Esta misma encuesta mostró que las mujeres en Filipinas también experimentaron una mayor pérdida que los hombres respecto a otras fuentes de ingresos, como las remesas, los ahorros y los negocios familiares. Con las pérdidas de empleo en el extranjero para las trabajadoras filipinas migrantes (y se utiliza el femenino porque son predominantemente mujeres), la economía en su conjunto sufrió una reducción de entre el 14 y el 20 por ciento en las entradas de remesas (Murakami, Shimizutani y Yamada 2021).

La pandemia está poniendo de manifiesto algunas de las fallas en cuanto a igualdad de género de Filipinas existentes antes de la pandemia que determinarán la recuperación del país a medio y largo plazo. Las normas sociales sobre las funciones reproductivas de las mujeres afectan a su capacidad para participar activamente en el mercado laboral y son uno de los factores que explican por qué Filipinas tenía la tasa de participación laboral femenina más baja de la región de Asia y el Pacífico. Según un análisis realizado para la Autoridad Nacional de Economía y Desarrollo (National Economic and Development Authority, NEDA), la tasa de participación laboral de las mujeres en Filipinas (46 por ciento) era la más baja de todos los países de la ASEAN antes de la pandemia; además, las mujeres casadas tenían 12 puntos porcentuales menos de probabilidades de estar ocupadas que las solteras, y las diferencias eran mayores para las mujeres con hijos pequeños (Cabegin y Gaddi 2019). Los roles tradicionales de género y las expectativas sociales sobre las responsabilidades de mujeres y hombres –las de cuidados para ellas y las de sostén de la familia para ellos– hacen que un mayor número de mujeres abandonen el mercado laboral tras el matrimonio o el nacimiento de los hijos.

Otros problemas de salud sexual y reproductiva de las mujeres se asocian a un mayor riesgo de exacerbar las brechas de género. Antes de la pandemia de COVID-19, Filipinas ya tenía una de las tasas más altas de embarazos en adolescentes (5 por ciento) entre los países asiáticos, y la mitad de las mujeres solteras en edad reproductiva declaraban una ausencia de servicios de planificación familiar (PSA e ICF 2018). Según las estimaciones disponibles, hubo 751 000 embarazos no deseados adicionales en 2020 debido a la reducción del acceso a los servicios de salud sexual y reproductiva (Marquez et al. 2020). El aumento de las tasas de natalidad en Filipinas durante la pandemia también contribuirá a incrementar el trabajo reproductivo de las mujeres (Johnson, Simonette y Drury

2020). En el contexto de la pandemia de COVID-19 y de la recesión económica concomitante, cabe esperar que estas tendencias interrelacionadas en los ámbitos del trabajo, las normas sociales y la salud sexual y reproductiva empeoren, con implicaciones a largo plazo para el empoderamiento económico de las mujeres y la igualdad de género.

## 3. Modelo de simulación

Analizamos la distribución por sexos de los efectos de la pandemia y de los confinamientos simulando cómo las perturbaciones de la demanda y la oferta asociadas se traducen en pérdidas de empleo y variaciones de los ingresos de las mujeres frente a los de los hombres. Así, nuestro modelo da cuenta tanto de las pérdidas causadas por las restricciones como de las que se producirían de todos modos debido al miedo al virus.[6] Como hicieron Del Rio-Chanona et al. (2020), nuestro enfoque de simulación estima los efectos de las perturbaciones de primer orden de la demanda y de la oferta que reflejan los impactos directos e inmediatos de las políticas de contención y de los cambios de comportamiento para evitar la exposición al virus. No predecimos los impactos generales, que incluirían los efectos de seguimiento de una mayor demanda y de las reducciones de la oferta, así como los efectos indirectos de los cambios en la cadena de suministro mundial.[7]

En esta sección describimos la simulación de los efectos en el empleo debidos a perturbaciones de la demanda y de la oferta. A continuación mostramos cómo estas perturbaciones se traducen en reducciones en términos de ingresos o de valor añadido. Dichas reducciones, a su vez, se utilizan para simular el efecto de la pandemia y del confinamiento en los niveles de pobreza.

### 3.1. Perturbaciones de la demanda

La presencia de la COVID-19 está teniendo una gran influencia en los patrones de gasto de los consumidores, incluso en ausencia de restricciones a la actividad comercial. Además de incrementar la demanda de servicios sanitarios, es probable que los consumidores intenten reducir su riesgo de exposición al virus y con ello disminuya su demanda de productos y servicios que impliquen un contacto estrecho con otras personas. Para incorporar estas pautas de comportamiento a nuestro modelo, seguimos el enfoque de Del Rio-Chanona et al. (2020) y recurrimos a las estimaciones desarrolladas por la Oficina Presupuestaria del Congreso de Los Estados Unidos (CBO 2006) para predecir el impacto potencial de una pandemia de gripe. La pandemia simulada por la CBO se basa vagamente en el brote de SARS-CoV-1 (síndrome respiratorio agudo grave por coronavirus) y trata de determinar las respuestas de comportamiento que la gente adoptaría para evitar la infección en cada sector de actividad económica. El análisis de la CBO supone que la demanda de bienes y servicios que conllevan riesgos de transmi-

[6] El análisis excluye los efectos de las políticas fiscales, como los subsidios en la fase inicial de la pandemia en el marco del Programa de Mejora Social (Social Amelioration Program).

[7] Para captar estos efectos secundarios se necesitaría un modelo de equilibrio general que también pudiera incorporar supuestos como el retorno de las trabajadoras filipinas emigradas.

sión, como el ocio y los viajes, se reduce debido al deseo de los consumidores de evitar la infección. La CBO elabora dos escenarios, uno grave y otro leve, y predice las perturbaciones de la demanda por sector en uno y otro basándose en opiniones de expertos. Nuestra decisión de adoptar el modelo econométrico de la CBO se apoya en la hipótesis de que, si bien la estructura de la economía de Filipinas difiere de la de los Estados Unidos en términos de peso sectorial, es probable que los comportamientos de los consumidores que determinan cambios en la demanda de productos y servicios específicos sean similares. Para llevar a cabo este análisis, reproducimos las estimaciones de la CBO utilizadas por Del Rio-Chanona et al. (2020) aplicándolas a los sectores económicos de la Clasificación Industrial Estándar de Filipinas (Philippine Standard Industrial Classification, PSIC).

Suponemos que los consumidores filipinos reaccionaron inicialmente a la presencia de la COVID-19 según el escenario de perturbación grave y que, de mediados de marzo de 2020 hasta finales de dicho año, se fueron desensibilizando a la presencia de la COVID-19, de forma que las perturbaciones fueron perdiendo gravedad a medida que pasaba la «ola».

### 3.2. Perturbaciones de la oferta

Las restricciones a la oferta de mano de obra se aplican en distinto grado dependiendo del sector, de la región administrativa y del momento. Se derivan de las exigencias de distanciamiento social y de los cierres de sectores de riesgo impuestos por las políticas de contención. El distanciamiento social se exige durante los periodos de cuarentena comunitaria en función de los porcentajes de trabajadores que el Grupo de Trabajo Interinstitucional (Inter-agency Task Force) permite en cada lugar de trabajo. Los trabajadores que no pueden acudir al lugar de trabajo no se convierten necesariamente en improductivos si pueden trabajar desde el domicilio, pero esto depende de las características de su lugar de domicilio (por ejemplo, si tienen un espacio de trabajo adecuado o acceso a un ordenador) y de la voluntad o capacidad del empleador para recurrir a modalidades de teletrabajo. A fin de reflejar esta situación en las estimaciones, además de los trabajadores permitidos en el lugar de trabajo, suponemos que el 50 por ciento de los trabajadores con ocupaciones que les permiten trabajar desde el domicilio lo hacen. Las reducciones relativas simuladas se basan en el grado de confinamiento comunitario aplicado en cada región administrativa y en cada momento. Se considera que las reducciones de mano de obra por sector de cada región administrativa están asociadas a la política de cuarentena comunitaria en vigor para el momento de 2020 analizado. Dado que las políticas de cuarentena comunitaria restringen principalmente el número de trabajadores permitidos en el lugar de trabajo, las políticas se simulan como perturbaciones de la oferta de mano de obra.

Como país en desarrollo, gran parte del producto de Filipinas se genera por el trabajo por cuenta propia y por el empleo informal. Para captar los efectos a través de estos canales, es necesario simular los efectos sobre el valor añadido, $Y$, a fin de reflejar la reducción de la demanda de mano de obra, $L$. Los efectos de las perturbaciones de la oferta se simulan mediante una función de producción, en la que se considera que la perturbación es una perturbación del insumo de trabajo. El producto proporcional del sector $i$ en la región administrativa $j$ en un momento posterior a la perturbación se toma como la proporción de trabajo restante, elevada a la potencia $\alpha$ (elasticidad del producto), que se aproxima como la proporción de trabajo correspondiente al producto del sector $i$ en la región administrativa $j$, como sigue:

$$\left(\frac{Y_{ij} - \Delta Y_{ij}}{Y_{ij}}\right) = \left(1 - \frac{\Delta L_{ij}}{L_{ij}}\right)^{\alpha_{ij}} \qquad (1)$$

La proporción de trabajo sectorial se aproxima como la remuneración total de los trabajadores del sector (coste salarial más prestaciones e impuestos), dividida por el valor del producto del sector, tras los impuestos sobre el valor añadido. El modelo se estima utilizando los microdatos de la LFS y la FIES de Filipinas, y se utilizan ponderaciones de la muestra para calcular la remuneración total por sector. La reducción del valor añadido debida a la COVID-19 se toma como la reducción proporcional multiplicada por el valor añadido del sector $i$ en la región administrativa $j$ en 2019, prorrateado a la parte del año analizada.

En la Contabilidad Nacional, el valor añadido se publica agregado según las categorías sectoriales de dos dígitos de la PSIC a escala nacional, y según las de un dígito a escala regional. Los microdatos de la LFS y de la FIES permiten calcular la distribución de los trabajadores dentro de las categorías sectoriales de un dígito a partir de las de dos dígitos por región administrativa. Para obtener el valor añadido por región administrativa agregado según las categorías de dos dígitos de la PSIC, se supone que el valor añadido dentro de cada categoría de un dígito sigue la distribución de la mano de obra en las categorías de dos dígitos en cada región administrativa multiplicada por la producción media nacional de los trabajadores de dicho sector.

### 3.3. Perturbaciones netas

Si las pérdidas de valor añadido debidas a las perturbaciones de la oferta se toman como las pérdidas observadas después de las restricciones a los trabajadores (cierres) y se combinan con las pérdidas derivadas de las reducciones de la demanda, obtendremos valores sobrestimados, dado que las reducciones de la demanda pueden haber provocado de todos modos una reducción de la producción del sector y la región administrativa en el momento dado. Por ejemplo, si las actividades turísticas no están permitidas y no hay demanda para ellas, la reducción del valor añadido del turismo que se produciría es del 100 por ciento, y la estimada, del 200 por ciento. Para evitar este tipo de doble contabilización, para un sector, una región administrativa y un momento determinados, se toma como reducción del valor añadido la mayor de las perturbaciones, ya sea de oferta o de demanda. Se trata de un enfoque conservador en cuanto al cálculo de las pérdidas, ya que los efectos sobre la oferta y la demanda se toman como sustitutos, y no combinados, mientras que las pérdidas reales pueden ser aditivas hasta cierto punto, en lugar de sustitutivas. Las variaciones en cada momento y región administrativa se prorratean a la parte del año correspondiente al momento analizado y luego se suman para obtener los cambios anuales por región administrativa, que a su vez se suman para obtener los cambios nacionales.

### 3.4. Desglose por género y por grupo de ingresos

Nuestro objetivo es comprender cómo se distribuyen los costes de las medidas de confinamiento según el sexo y el grupo de ingresos. Para ello, es necesario simular las perturbaciones de los ingresos. En el caso de los ingresos salariales, este proceso es sencillo: las reducciones de la demanda de mano de obra equivalen a reducciones de los ingresos, suponiendo la libertad del empleador de despedir o ajustar el número de horas de trabajo de la mayoría de los empleados, y que las prestaciones por desempleo no están muy extendidas. En el caso de los trabajadores por cuenta propia o de los propietarios de empresas, las reducciones del valor añadido equivalen a reducciones de los ingresos. A continuación, se multiplica la proporción de trabajadores asalariados de cada sector y región administrativa por la reducción estimada de mano de obra, y la proporción de trabajadores no asalariados, por la reducción estimada del valor añadido. Estos valores se suman para obtener la variación proporcional de la renta por el lado de la oferta.

Por el lado de la demanda, las reducciones de la demanda de producto se contabilizan como reducciones de la demanda de mano de obra mediante una función de producción de Cobb-Douglas:

$$\frac{\Delta L_{ij}}{L_{ij}} = \left(1 - \frac{\delta_{ij}}{Y_{ij}}\right)^{\frac{1}{\alpha_{ij}}} \tag{2}$$

donde la proporción de producto que sigue generándose se eleva a la inversa de la proporción de trabajo, y el término $\delta/Y$ representa la reducción proporcional del valor añadido. Para cada momento, sector y región administrativa se toma la mayor de las reducciones proporcionales de la renta inducidas por la demanda o por la oferta. A continuación, las reducciones de cada momento se prorratean en función de la parte del año correspondiente y se suman para obtener los cambios proporcionales anuales.

Para traducir los cambios proporcionales en cambios absolutos en el empleo, los primeros se multiplican por el número de trabajadores y trabajadoras en cada categoría sectorial de la PSIC y cada región administrativa y luego se suman.

En el caso de los ingresos, los cambios proporcionales se multiplican por los ingresos de los hogares ponderados para cada sector y región administrativa en los microdatos de la FIES de 2015. Estos ingresos se restan en una proporción uniforme entre todos los trabajadores del sector y la región administrativa, ya que no es posible determinar qué trabajadores dentro de los mismos tendrían más probabilidades de perder su empleo. Este ejercicio se repite para los ingresos femeninos y masculinos por sector y región administrativa, para captar los efectos diferenciados por género de las restricciones sectoriales y regionales, basándose en la proporción de empleo e ingresos por género en cada sector y región administrativa.

Para calcular los efectos sobre el nivel de pobreza, se agregan los ingresos después de los ajustes a nivel de los hogares utilizando los microdatos de la FIES de 2015 y se ponderan para reflejar las poblaciones de hogares a escala provincial y nacional. A continuación, se contabiliza el número de hogares que caen por debajo de los umbrales de pobreza provinciales de 2015 y de otros indicadores de pobreza, y se comparan con los valores reales de 2015 utilizando una serie de agregaciones para explorar los efectos en diferentes subpoblaciones de interés.

### 4. Resultados

Los resultados de la estimación de la ecuación (1) indican una reducción sustancial de la actividad económica, con un descenso del 13,5 por ciento a nivel nacional (cuadro 1). Las pérdidas de actividad económica se concentran tanto en las zonas más urbanas, sometidas a medidas de confinamiento más largas e intensas, como en las zonas dependientes de sectores muy afectados, como el turismo. Observamos que esta reducción simulada de la actividad económica es similar a la disminución real del crecimiento del PIB, que cayó 15,5 puntos porcentuales, pasando del 6 por ciento en 2019 al –9,5 por ciento en 2020. Dado que la disminución simulada es ligeramente inferior a los valores reales, los resultados presentados pueden subestimar ligeramente los efectos de la pandemia.

Los efectos estimados sobre la demanda de mano de obra y el empleo son aún más intensos que los efectos sobre el valor añadido y difieren sustancialmente según el sexo (cuadro 2). Nuestra simulación apunta a una reducción de la mano de obra un 20 por ciento mayor para las mujeres en comparación con los hombres, lo que refleja la mayor proporción de mano de obra femenina en

**Cuadro 1. Reducción simulada de la actividad económica en Filipinas como consecuencia de la COVID-19 y de las medidas de contención asociadas, por región administrativa (en millones de pesos filipinos)**

| Región administrativa | 2019 (ref.) | Valor añadido perdido, 2020 | Valor añadido perdido, 2020 (%) |
|---|---|---|---|
| Región I (Región de Ilocos) | 650 542 | 95 187 | 14,6 |
| Región II (Valle de Cagayán) | 396 522 | 52 317 | 13,2 |
| Región III (Luzón Central) | 2 173 084 | 305 165 | 14,0 |
| Región IV-A (Calabarzón) | 2 855 689 | 400 795 | 14,0 |
| Región de Tagala Sudoeste (Mimaropa) | 376 103 | 54 043 | 14,4 |
| Región V (Bícol) | 556 981 | 78 335 | 14,1 |
| Región VI (Bisayas Occidentales) | 912 135 | 142 501 | 15,6 |
| Región VII (Bisayas Centrales) | 1 262 484 | 239 350 | 19,0 |
| Región VIII (Bisayas Orientales) | 463 608 | 46 392 | 10,0 |
| Región IX (Península de Zamboanga) | 395 042 | 42 810 | 10,8 |
| Región X (Mindanao del Norte) | 877 080 | 75 570 | 8,6 |
| Región XI (Davao) | 919 092 | 114 284 | 12,4 |
| Región XII (Mindanao Central, Soccsksargen) | 467 502 | 49 821 | 10,7 |
| Región de la Capital Nacional (NCR) | 6 235 408 | 816 224 | 13,1 |
| Región Administrativa de la Cordillera (CAR) | 320 695 | 42 926 | 13,4 |
| Nación Mora (Bangsamoro) | 244 840 | 24 960 | 10,2 |
| Región XIII (Caraga) | 304 341 | 32 918 | 10,8 |
| **Total** | **19 411 148** | **2 613 598** | **13,5** |

Notas: Denominaciones de las regiones administrativas según los códigos geográficos normalizados de Filipinas (Philippine Statistics Authority) al 30 de junio de 2021. Tipo de cambio promedio para 2019-2020: 1 dólar de los Estados Unidos = 50,71 pesos filipinos (Bangko Sentral ng Pilipinas).

Fuentes: Cálculos de los autores con datos de la LFS y de la FIES de Filipinas.

#### Cuadro 2. Reducción simulada de la demanda de mano de obra en Filipinas como consecuencia de la COVID-19 y de las medidas de contención adoptadas, por sexo

| Región administrativa | Empleo en 2019 (ref.) | | Reducción de la demanda de mano de obra, 2020 | | Reducción del empleo, 2020 (%) | |
|---|---|---|---|---|---|---|
| | Hombres | Mujeres | Hombres | Mujeres | Hombres | Mujeres |
| Región I (Región de Ilocos) | 1 306 665 | 778 096 | 279 273 | 200 453 | 21,4 | 25,8 |
| Región II (Valle de Cagayán) | 957 307 | 551 377 | 155 408 | 121 503 | 16,2 | 22,0 |
| Región III (Luzón Central) | 2 634 589 | 1 654 403 | 723 220 | 518 487 | 27,5 | 31,3 |
| Región IV-A (Calabarzón) | 2 985 655 | 2 243 622 | 857 668 | 717 266 | 28,7 | 32,0 |
| Región Tagala Sudoeste (Mimaropa) | 806 309 | 496 966 | 141 971 | 118 502 | 17,6 | 23,8 |
| Región V (Bícol) | 1 473 980 | 901 111 | 279 026 | 230 561 | 18,9 | 25,6 |
| Región VI (Bisayas Occidentales) | 1 971 874 | 1 324 941 | 382 150 | 330 436 | 19,4 | 24,9 |
| Región VII (Bisayas Centrales) | 1 781 313 | 1 364 460 | 492 640 | 420 389 | 27,7 | 30,8 |
| Región VIII (Bisayas Orientales) | 1 129 613 | 683 429 | 173 071 | 135 737 | 15,3 | 19,9 |
| Región IX (Península de Zamboanga) | 890 688 | 524 300 | 140 064 | 103 945 | 15,7 | 19,8 |
| Región X (Mindanao del Norte) | 1 182 715 | 744 754 | 208 621 | 143 857 | 17,6 | 19,3 |
| Región XI (Davao) | 1 229 454 | 716 435 | 257 032 | 179 461 | 20,9 | 25,0 |
| Región XII (Mindanao Central, Soccsksargen) | 1 076 855 | 658 210 | 167 725 | 132 760 | 15,6 | 20,2 |
| Región de la Capital Nacional (NCR) | 2 709 564 | 2 223 586 | 887 634 | 742 440 | 32,8 | 33,4 |
| Región Administrativa de la Cordillera (CAR) | 458 424 | 306 969 | 80 021 | 65 878 | 17,5 | 21,5 |
| Nación Mora (Bangsamoro) | 889 397 | 329 524 | 100 601 | 49 995 | 11,3 | 15,2 |
| Región XIII (Caraga) | 669 929 | 395 233 | 110 759 | 82 801 | 16,5 | 21,0 |
| **Total** | **24 154 330** | **15 897 418** | **5 436 882** | **4 294 471** | **22,5** | **27,0** |

Nota: Denominaciones de las regiones administrativas según los códigos geográficos normalizados de Filipinas (Philippine Statistics Authority) al 30 de junio de 2021.

Fuentes: Cálculos de los autores con datos de la LFS y de la FIES de Filipinas.

algunos de los sectores que han sufrido las mayores pérdidas, como las agencias de viajes y los servicios de empleo. En general, las mujeres experimentan un descenso del 27 por ciento en el empleo, en comparación con el 22,5 por ciento de los hombres. En todas las regiones de Filipinas se observan mayores reducciones relativas de la demanda de mano de obra femenina.

Nuestras estimaciones también permiten observar que las reducciones de la demanda de mano de obra se traducen en efectos sustanciales sobre los ingresos de los hogares en 2020, con pérdidas del 29,1 por ciento de los ingresos generados por las trabajadoras y del 26,2 por ciento de los ingresos generados por los trabajadores (cuadro 3), cifras mucho más elevadas que la correspondiente a la reducción del valor añadido nacional simulado, del 13,5 por ciento. Por lo tanto, la reducción relativa de las mujeres es más de un 10 por ciento mayor que la de los hombres. Este patrón nacional se repite en todas las regiones administrativas, excepto en la Región de la Capital Nacional, donde las reducciones relativas son casi iguales entre hombres y mujeres. En términos absolutos, las pérdidas de los hombres son mayores que las de las mujeres, debido en gran medida a los mayores salarios y a la mayor participación laboral masculina antes de la pandemia de COVID-19.

Estas pérdidas de ingresos afectan más a las personas pobres y vulnerables. Se calcula que la pobreza casi se duplica en Filipinas con respecto al año de referencia sin COVID-19, con un aumento del 73,3 por ciento a nivel nacional bajo las políticas de contención adoptadas en 2020 (cuadro 4). Este aumento

### Cuadro 3. Reducción simulada de los ingresos de los hogares en Filipinas como consecuencia de la COVID-19 y de las medidas de contención asociadas (en millones de pesos filipinos)

| Región | Ingresos en 2019 (ref.) | | Pérdidas en 2020 | | Pérdidas en 2020 (%) | |
|---|---|---|---|---|---|---|
| | Hombres | Mujeres | Hombres | Mujeres | Hombres | Mujeres |
| Región I (Región de Ilocos) | 105071 | 58869 | 25905 | 15910 | 24,7 | 27,0 |
| Región II (Valle de Cagayán) | 69992 | 38232 | 14297 | 10510 | 20,4 | 27,5 |
| Región III (Luzón Central) | 284726 | 170700 | 85040 | 55117 | 29,9 | 32,3 |
| Región IV-A (Calabarzón) | 348954 | 235167 | 103655 | 72841 | 29,7 | 31,0 |
| Región de Tagala Sudoeste (Mimaropa) | 55603 | 33664 | 11345 | 9683 | 20,4 | 28,8 |
| Región V (Bícol) | 104211 | 66988 | 23238 | 19256 | 22,3 | 28,7 |
| Región VI (Bisayas Occidentales) | 139986 | 100753 | 31413 | 27073 | 22,4 | 26,9 |
| Región VII (Bisayas Centrales) | 154220 | 109405 | 49135 | 37607 | 31,9 | 34,4 |
| Región VIII (Bisayas Orientales) | 80575 | 53618 | 14350 | 11725 | 17,8 | 21,9 |
| Región IX (Península de Zamboanga) | 63450 | 36863 | 12118 | 9087 | 19,1 | 24,7 |
| Región X (Mindanao del Norte) | 96046 | 56769 | 18847 | 12671 | 19,6 | 22,3 |
| Región XI (Davao) | 108506 | 67385 | 24682 | 17321 | 22,7 | 25,7 |
| Región XII (Mindanao Central, Soccsksargen) | 71579 | 47866 | 12894 | 10964 | 18,0 | 22,9 |
| Región de la Capital Nacional (NCR) | 447074 | 324022 | 143146 | 101761 | 32,0 | 31,4 |
| Región Administrativa de la Cordillera (CAR) | 41539 | 28345 | 9189 | 7386 | 22,1 | 26,1 |
| Nación Mora (Bangsamoro) | 49254 | 18093 | 6643 | 4153 | 13,5 | 23,0 |
| Región XIII (Caraga) | 54582 | 34587 | 10691 | 8209 | 19,6 | 23,7 |
| **Total** | **2275366** | **1481327** | **596589** | **431274** | **26,2** | **29,1** |

Notas: Denominaciones de las regiones administrativas según los códigos geográficos normalizados de Filipinas (Philippine Statistics Authority) al 30 de junio de 2021. Tipo de cambio promedio para 2019-2020: 1 dólar de los Estados Unidos = 50,71 pesos filipinos (Bangko Sentral ng Pilipinas).

Fuentes: Cálculos de los autores con datos de la LFS y de la FIES de Filipinas.

### Cuadro 4. Variación simulada de los niveles de pobreza en Filipinas como consecuencia de la COVID-19 y de las medidas de contención asociadas, por tipo de hogar

| Tipo de hogar | | Tasa de pobreza relativa (%) | Desigualdad de ingresos | |
|---|---|---|---|---|
| | | | Gini | P75/P25 |
| Todos | Solo por efecto de la pandemia | 140,4 | 103,5 | 102,5 |
| | Medidas de contención | 173,3 | 100,1 | 94,6 |
| Urbanos | Solo por efecto de la pandemia | 174,9 | 105,3 | 108,4 |
| | Medidas de contención | 237,2 | 103,9 | 102,5 |
| Rurales | Solo por efecto de la pandemia | 131,2 | 103,1 | 101,0 |
| | Medidas de contención | 156,2 | 98,3 | 96,0 |
| Mujer cabeza de familia | Solo por efecto de la pandemia | 147,4 | 102,0 | 102,6 |
| | Medidas de contención | 190,4 | 102,1 | 99,9 |
| Hombre cabeza de familia | Solo por efecto de la pandemia | 139,3 | 102,5 | 102,0 |
| | Medidas de contención | 170,7 | 97,9 | 94,3 |
| Urbanos con mujer cabeza de familia | Solo por efecto de la pandemia | 168,7 | 105,2 | 106,6 |
| | Medidas de contención | 232,0 | 106,9 | 105,4 |
| Urbanos con hombre cabeza de familia | Solo por efecto de la pandemia | 176,2 | 104,6 | 109,8 |
| | Medidas de contención | 238,3 | 101,8 | 102,5 |
| Rurales con mujer cabeza de familia | Solo por efecto de la pandemia | 138,0 | 102,0 | 101,0 |
| | Medidas de contención | 171,9 | 100,5 | 96,8 |
| Rurales con hombre cabeza de familia | Solo por efecto de la pandemia | 130,3 | 103,6 | 101,0 |
| | Medidas de contención | 154,1 | 97,7 | 95,7 |

Nota: El indicador P75/P25 corresponde a la ratio entre los ingresos salariales de los trabajadores en los percentiles 75.º y 25.º.

Fuentes: Cálculos de los autores con datos de la LFS y de la FIES de Filipinas.

de la pobreza es más pronunciado en las zonas urbanas que en las rurales, a diferencia de lo ocurrido en muchas otras crisis económicas en el pasado. Parte de este impacto desproporcionado en las zonas urbanas se debe a las industrias afectadas por los cambios en la demanda impulsados por la pandemia, pero la principal explicación es la concentración de las medidas de contención en las zonas más urbanizadas. Los hogares encabezados por mujeres se ven mucho más afectados que los encabezados por hombres, pero esta disparidad se produce principalmente en las zonas rurales, no en las urbanas. Incluso en ausencia de políticas de contención, las perturbaciones de la demanda afectan más al nivel de pobreza de los hogares encabezados por mujeres que al de los encabezados por hombres, pero las políticas de contención amplían la disparidad.

### 5. Cierre de escuelas: costes a corto plazo para docentes y progenitores

El cierre de los centros escolares tiene consecuencias adicionales en el mercado laboral, en términos de pérdidas tanto de empleo del personal docente de escuelas privadas como de ingresos para los padres y madres que tienen que hacerse cargo de los niños. La matrícula de las escuelas privadas se redujo en un 48 por ciento en el año escolar 2020-2021, según las cifras del Ministerio de Educación de septiembre de 2020 (Luz 2020). Según el Consejo Coordinador de Asociaciones Educativas Privadas, las escuelas privadas de Filipinas emplean a 409 757 docentes y otros trabajadores (Magsambol 2020). Los datos de la LFS indican que las mujeres representan dos tercios del total del personal docente. Basándonos en los datos fusionados de la FIES y la LFS, suponemos que el 30 por ciento de esas maestras y maestros pierden su empleo. Ello equivale a la pérdida de aproximadamente 123 000 puestos de trabajo en el curso escolar 2020-2021, con una pérdida salarial estimada de 16 000 millones de pesos filipinos.

En cuanto a las pérdidas de ingresos de padres y madres, utilizamos los datos fusionados de la FIES y la LFS de 2015 para identificar todos los hogares con niños menores de 12 años cuyos progenitores trabajaban. Suponemos que un adulto debe haber cursado al menos educación secundaria para poder ayudar al aprendizaje en línea de los niños. También suponemos que en los hogares en los que ambos progenitores trabajaban y en los que no había abuelos menores de 80 años con al menos educación secundaria, uno de los progenitores tendría que quedarse en casa y sería, por consiguiente, el que perdería más ingresos. En esos hogares, aproximadamente el 92 por ciento de los padres ganan más que las madres. Sin embargo, las madres tienen una escolaridad media de 9,5 años, frente a los 8,5 años de los padres. La combinación de unos ingresos más bajos y una mayor escolaridad convierte a las madres en el progenitor con más probabilidades de dejar de trabajar para ayudar al aprendizaje en línea de los niños. Aproximadamente un tercio de los hogares de la muestra tienen estas características. Nuestra simulación apunta a una pérdida total proyectada del 6 por ciento de los ingresos de los hogares, lo que equivale a 225 000 millones de pesos para un año escolar. La mayor parte de esta pérdida (207 000 millones de pesos) refleja la pérdida de ingresos de las mujeres por quedarse en casa para cuidar a los niños y educar en el hogar, por lo que los ingresos de las mujeres disminuyen un 14 por ciento, frente a los de los hombres, que disminuyen un 0,1 por ciento. A esto se unen los salarios perdidos de los docentes de escuelas privadas debido a los cierres de escuelas, a tenor de 10 700 millones de pesos para las maestras y 5300 millones de pesos para los maestros.

A medio y largo plazo, el cierre prolongado de las escuelas presenciales amenaza la capacidad de los jóvenes del país para convertirse en trabajadores productivos, y este riesgo recae con más fuerza en los hogares más pobres, que son los que más dificultades tienen para seguir las modalidades de aprendizaje a distancia. El porcentaje de niños que han dejado de ir a la escuela en Filipinas es mayor que en otros países de la región, al igual que un porcentaje relativamente bajo de niños (21 por ciento) ha asistido a clases en línea (si se han ofrecido) debido a las inestables conexiones a internet y a la falta de dispositivos digitales (Morgan y Trinh 2021). A juzgar por las mayores tasas de abandono escolar de las niñas en Filipinas durante la crisis financiera asiática de 1998-1999, es posible que las niñas no vuelvan a la escuela tan rápidamente como los niños tras el cierre de los centros escolares por la COVID-19 (Banco Mundial 2020). La pérdida de puestos de trabajo para las mujeres y las mayores tasas de abandono escolar de las niñas, junto con el aumento de los embarazos no deseados de los que informan los medios de comunicación, no solo harán retroceder los avances en materia de igualdad de género, sino que también podrían frenar los esfuerzos de recuperación económica, al reducir las oportunidades de trabajo cualificado para una parte importante de la población.

### 6. Conclusión y recomendaciones de políticas

La pandemia de COVID-19 y las políticas de contención asociadas han restringido enormemente en Filipinas la capacidad de los trabajadores y, sobre todo, de las trabajadoras para participar en el mercado laboral y contribuir a la economía. La pandemia ha puesto de manifiesto la vulnerabilidad de las personas más pobres, en particular, nuevamente, las mujeres, ante la crisis económica, y los resultados de nuestro ejercicio de simulación apuntan a que las personas de bajos ingresos están soportando relativamente más sus costes en Filipinas. La pandemia también está poniendo de manifiesto las fallas preexistentes del sistema (normas sociales, servicios, infraestructuras) y, en particular, las disparidades en cuanto a las experiencias vividas por hombres y mujeres y por los diferentes grupos de ingresos. Nuestro análisis de simulación predice grandes aumentos del nivel de pobreza a corto plazo que podrían tardar años en superarse. Estos efectos afectarán a su vez a las estrategias de recuperación.

En el momento de redactar el presente artículo se preveía que las políticas de confinamiento causantes de muchas de estas pérdidas continuaran en 2021. Sin embargo, las pérdidas observadas con nuestro análisis no tenían por qué continuar. Existen alternativas a los confinamientos y a los cierres de escuelas con los que se puede lograr un mayor control de la pandemia sin sacrificar el bienestar futuro de las mujeres y de las personas más pobres. Por ejemplo, Chen et al. (2020) observan grandes resultados con medidas tales como el distanciamiento social, el rastreo de contactos y las bajas por enfermedad remuneradas, y algo menores con los cierres de escuelas y las restricciones de movimiento.

Recurrir más a estos enfoques puede ayudar a reducir las pérdidas que sufren los grupos desfavorecidos.

Dadas las consecuencias adversas, directas e indirectas, de la pandemia y de las medidas de confinamiento sobre la igualdad de género en Filipinas, las estrategias de recuperación deben tener en cuenta cómo se interrelacionan las normas sociales tradicionales y las desigualdades de género, con el fin de promover una recuperación más equitativa. Aunque Filipinas ha hecho grandes progresos en materia de igualdad de género en las últimas décadas, las expectativas de que las mujeres deben cumplir con los roles reproductivos tradicionales son un determinante de su baja participación laboral y de su particular vulnerabilidad a la pobreza en el contexto actual de pandemia. El Gobierno de Filipinas ha sido proactivo en cuanto a la provisión de cobertura de seguro médico para la COVID-19, así como en cuanto a las transferencias en efectivo y los subsidios alimentarios para los hogares pobres (Park e Inocencio 2020). Dado que la COVID-19 pone en peligro los avances logrados en materia de igualdad de género, la estrategia política del Gobierno debe poner aún más énfasis en una recuperación impulsada por los cuidados, con una generosa protección social, medidas anticíclicas para generar demanda agregada e inversiones en sistemas de cuidados vitales. La inversión pública en servicios de atención a la infancia y de cuidados de larga duración puede crear millones de puestos de trabajo, tanto directamente en el sector de los cuidados, como indirectamente a través de la actividad económica inducida en algunos de los sectores más afectados, como el comercio minorista, la asistencia sanitaria y los servicios de alimentación, que emplean de forma desproporcionada a mujeres. Los efectos de las medidas de confinamiento adoptadas hasta ahora deberán mitigarse, entre otras cosas, mediante un apoyo integral que permita a las mujeres acceder a un trabajo decente y a oportunidades de mejora de sus capacidades, incluida la ampliación de la oferta de guarderías asequibles y de buena calidad. También son cruciales los esfuerzos dedicados a que las niñas vuelvan a la escuela, dada su probabilidad relativamente más alta de abandono.

Garantizar que las mujeres y las niñas tengan a disposición servicios de salud reproductiva y sexual como parte de la respuesta a la pandemia y de la estrategia de recuperación es igualmente vital para reducir el elevado número de embarazos no deseados y de matrimonios precoces, así como para proteger la salud materna e infantil. Los embarazos de adolescentes y no deseados no solo están relacionados con peores niveles de salud en las mujeres y sus hijos, sino que también es probable que agraven la vulnerabilidad de las mujeres frente a la pobreza y reduzcan sus oportunidades de entrar en el mercado laboral o de continuar estudiando. La Comisión de Población (Commission on Population) de Filipinas ha reconocido la importancia de abordar el problema de los embarazos de adolescentes durante la pandemia y ha puesto en marcha nuevas iniciativas destinadas a movilizar y a concienciar a las administraciones locales, los proveedores de servicios sanitarios y las comunidades. Será fundamental ampliar estas iniciativas. La dotación de los presupuestos públicos, la ampliación de los servicios de telesalud y la exploración de más vías para aumentar el acceso a la planificación familiar durante los confinamientos contribuirán a salvaguardar la salud y el bienestar de las mujeres y de sus hijos, ayudando así a contrarrestar los efectos negativos de los confinamientos en el empleo y en los ingresos de las mujeres que hemos documentado en nuestro estudio.

### Bibliografía citada

Alon, Titan, Matthias Doepke, Jane Olmstead-Rumsey y Michèle Tertilt. 2020. «The Impact of Covid-19 on Gender Equality». *COVID Economics Vetted and Real-Time Papers*, No. 4 (abril), 62-85.

Bahn, Kate, Jennifer Cohen e Yana van der Meulen Rodgers. 2020. «A Feminist Perspective on COVID-19 and the Value of Care Work Globally». *Gender, Work & Organization* 27 (5): 695-699.

Banco Mundial. 2020. COVID-19: *Impacto en la educación y respuestas de política pública*. Washington.

BAsD (Banco Asiático de Desarrollo). 2015. *Asian Development Outlook 2015 Update: Enabling Women, Energizing Asia*. Manila.

Cabegin, Emily Christi A., y Rebecca S. Gaddi. 2019. *Determinants of Female Labor Force Participation in the Philippines*. Pasig City: National Economic and Development Authority.

CBO (Congressional Budget Office), Estados Unidos. 2006. «A Potential Influenza Pandemic: Possible Macroeconomic Effects and Policy Issues». 27 de julio de 2006. Washington.

CESPAP (Comisión Económica y Social para Asia y el Pacífico). 2020. *The Covid-19 Pandemic and Violence Against Women in Asia and the Pacific*. Bangkok.

Chen, Liming, David Raitzer, Rana Hasan, Rouselle Lavado y Orlee Velarde. 2020. «What Works to Control COVID-19? Econometric Analysis of a Cross-Country Panel». ADB Economics Working Paper Series, No. 625. Manila: Banco Asiático de Desarrollo.

Cho, Yoonyoung, Jorge Avalos, Yasuhiro Kawasoe, Doug Johnson y Ruth Rodriguez. 2021. «The Impact of the COVID-19 Pandemic on Low Income Households in the Philippines: Impending Human Capital Crisis», COVID-19 Low Income HOPE Survey Note No. 3. Washington: Banco Mundial.

Craig, Lyn, y Brendan Churchill. 2021. «Working and Caring at Home: Gender Differences in the Effects of Covid-19 on Paid and Unpaid Labor in Australia». *Feminist Economics* 27 (1-2): 310-326.

De los Santos, Janet Alexis A., y Leodoro J. Labrague. 2021. «The Impact of Fear of COVID-19 on Job Stress, and Turnover Intentions of Frontline Nurses in the Community: A Cross-Sectional Study in the Philippines». *Traumatology* 27 (1): 52-59.

Del Rio-Chanona, R. Maria, Penny Mealy, Anton Pichler, François Lafond y J. Doyne Farmer. 2020. «Supply and Demand Shocks in the COVID-19 Pandemic: An Industry and Occupation Perspective». *Oxford Review of Economic Policy* 36 (S1): S94-S137.

FEM (Foro Económico Mundial). 2019. *Global Gender Gap Report 2020*. Ginebra.

— 2021. *Global Gender Gap Report 2021*. Ginebra.

Guadagno, Lorenzo. 2020. «Migrants and the COVID-19 Pandemic: An Initial Analysis», Migration Research Series, No. 60. Ginebra: OIM.

Hapal, Karl. 2021. «The Philippines' COVID-19 Response: Securitising the Pandemic and Disciplining the Pasaway». *Journal of Current Southeast Asian Affairs* 40 (2): 224-244.

İlkkaracan, İpek, y Emel Memiş. 2021. «Transformations in the Gender Gaps in Paid and Unpaid Work during the COVID-19 Pandemic: Findings from Turkey». *Feminist Economics* 27 (1-2): 288-309.

Johnson, Howard, Virma Simonette y Flora Drury. 2020. «Covid-19: The Philippines and Its Lockdown Baby Boom». *BBC News*, 23 de diciembre de 2020. https://www.bbc.com/news/world-asia-55299912.

Kabeer, Naila, Shahra Razavi e Yana van der Meulen Rodgers. 2021. «Feminist Economic Perspectives on the COVID-19 Pandemic». *Feminist Economics* 27 (1-2): 1-29.

Koh, David. 2020. «Migrant Workers and COVID-19». *Occupational and Environmental Medicine* 77 (9): 634-636.

Luz, Juan Miguel. 2020. «Too Many Children Not Enrolled this School Year Is a Major Concern». *Rappler.com*, 19 de noviembre de 2020. https://www.rappler.com/voices/ispeak/analysis-too-many-children-not-enrolled-this-school-year-major-concern/.

Magsambol, Bonz. 2020. «Over 400,000 Private School Employees Affected by Lockdown – Group». *Rappler.com*, 27 de abril de 2020. https://www.rappler.com/nation/259204-private-schools-affected-coronavirus-pandemic/.

Marquez, Maria Paz N., Elma P. Laguna, Maria Midea M. Kabamalan y Grace T. Cruz. 2020. «Estimating the Potential Impact of the COVID-19 Pandemic on Key Sexual and Reproductive Health Outcomes in the Philippines». Informe técnico preparado por el University of the Philippines Population Institute para el Fondo de Población de las Naciones Unidas. Manila: University of the Philippines Population Institute.

Morgan, Peter J., y Long Q. Trinh. 2021. «Impacts of COVID-19 on Households in ASEAN Countries and Their Implications for Human Capital Development». ADBI Working Paper Series, No. 1226. Tokyo: Asian Development Bank Institute.

Murakami, Enerelt, Satoshi Shimizutani y Eiji Yamada. 2021. «Projection of the Effects of the COVID-19 Pandemic on the Welfare of Remittance-Dependent Households in the Philippines». *Economics of Disasters and Climate Change* 5 (1): 97-110.

OIT. 2019. *El trabajo de cuidados y los trabajadores del cuidado para un futuro con trabajo decente*. Ginebra.

— 2020. «La respuesta al COVID-19: Lograr la igualdad de género en pos de un mejor futuro para las mujeres en el trabajo», Reseña de políticas, mayo de 2020. Ginebra.

— 2021. «Observatorio de la OIT: La COVID-19 y el mundo del trabajo». Séptima edición, 25 de enero de 2021. Ginebra.

ONU Mujeres y Women Count. 2020. *Unlocking the Lockdown: The Gendered Effects of COVID-19 on Achieving the SDGs in Asia and the Pacific*. Bangkok: Oficina Regional de las Naciones Unidas en Asia y el Pacífico.

Park, Cyn-Young, y Ancilla Marie Inocencio. 2020. «COVID-19 Is No Excuse to Regress on Gender Equality». ADB Briefs, No. 157. Manila: Banco Asiático de Desarrollo.

PNUD (Programa de las Naciones Unidas para el Desarrollo). 2020. *Informe sobre Desarrollo Humano 2020: La próxima frontera – El desarrollo humano y el Antropoceno*. Nueva York.

PSA (Philippine Statistics Authority). 2020. «Employment Situation in April 2020». 11 de noviembre de 2020. https://psa.gov.ph/statistics/survey/labor-and-employment/labor-force-survey/title/Employment%20Situation%20in%20April%202020.

PSA e ICF (Inner City Fund). 2018. *Philippines: National Demographic and Health Survey 2017*. Quezon City, Philippines, y Rockville.

Raitzer, David A., Rouselle F. Lavado, Jomar Rabajante, Xylee Javier, Ludigil Garces y Glenita Amoranto. 2020. «Cost–Benefit Analysis of Face-to-Face Closure of Schools to Control COVID-19 in the Philippines». ADB Briefs, No. 162. Manila: Banco Asiático de Desarrollo.

Roberton, Timothy, Emily D. Carter, Victoria B. Chou, Angela R. Stegmuller, Bianca D. Jackson, Yvonne Tam, Talata Sawadogo-Lewis y Neff Walker. 2020. «Early Estimates of the Indirect Effects of the COVID-19 Pandemic on Maternal and Child Mortality in Low-Income and Middle-Income Countries: A Modelling Study». *Lancet Global Health* 8 (7): E901-E908.

Sediri, Sabrine, Yosra Zgueb, Sami Ouanes, Uta Ouali, Soumaya Bourgou, Rabaa Jomli y Fethi Nacef. 2020. «Women's Mental Health: Acute Impact of COVID-19 Pandemic on Domestic Violence». *Archives of Women's Mental Health* 23 (6): 749-756.

Stevano, Sara, Alessandra Mezzadri, Lorena Lombardozzi y Hannah Bargawi. 2021. «Hidden Abodes in Plain Sight: The Social Reproduction of Households and Labor in the COVID-19 Pandemic». *Feminist Economics* 27 (1-2): 271-287.

Tee, Michael L., Cherica A. Tee, Joseph P. Anlacan, Katrina Joy G. Aligam, Patrick Wincy C. Reyes, Vipat Kuruchittham y Roger C. Ho. 2020. «Psychological Impact of COVID-19 Pandemic in the Philippines». *Journal of Affective Disorders* 277 (diciembre): 379-391.

UNESCO (Organización de las Naciones Unidas para la Educación, la Ciencia y la Cultura). 2020. «COVID-19: La UNESCO y sus asociados del sector de la educación presentan la campaña mundial "Las niñas en primer plano"», 28 de agosto de 2020. https://es.unesco.org/news/covid-19-unesco-y-sus-asociados-del-sector-educacion-presentan-campana-mundial-ninas-primer.

Wenham, Clare, Julia Smith y Rosemary Morgan. 2020. «COVID-19: The Gendered Impacts of the Outbreak». *Lancet* 395 (10227): 846-848.

Zamarro, Gema, y María J. Prados. 2021. «Gender Differences in Couples' Division of Childcare, Work and Mental Health during COVID-19». *Review of Economics of the Household* 19 (1): 11-40.

Zveglich, Joseph E., Jr., Yana van der Meulen Rodgers y Editha A. Laviña. 2019. «Expected Work Experience and the Gender Wage Gap: A New Human Capital Measure». *Economic Modelling* 83 (diciembre): 372-383.
